# Component-Based Design and Assembly of Heuristic Multiple Sequence Alignment Algorithms

**DOI:** 10.3389/fgene.2020.00105

**Published:** 2020-02-27

**Authors:** Haihe Shi, Xuchu Zhang

**Affiliations:** School of Computer and Information Engineering, Jiangxi Normal University, Nanchang, China

**Keywords:** heuristic multiple sequence alignment algorithms, feature model, generative programming, component interaction model, partition and recur platform

## Abstract

In recent years, there has been an explosive increase in the amount of bioinformatics data produced, but data are not information. The purpose of bioinformatics research is to obtain information with biological significance from large amounts of data. Multiple sequence alignment is widely used in sequence homology detection, protein secondary and tertiary structure prediction, phylogenetic tree analysis, and other fields. Existing research mainly focuses on the specific steps of the algorithm or on specific problems, and there is a lack of high-level abstract domain algorithm frameworks. As a result, multiple sequence alignment algorithms are complex, redundant, and difficult to understand, and it is not easy for users to select the appropriate algorithm, which may lead to computing errors. Here, through in-depth study and analysis of the heuristic multiple sequence alignment algorithm (HMSAA) domain, a domain-feature model and an interactive model of HMSAA components have been established according to the generative programming method. With the support of the PAR (partition and recur) platform, the HMSAA algorithm component library is formalized and a specific alignment algorithm is assembled, thus improving the reliability of algorithm assembly. This work provides a valuable theoretical reference for the applications of other biological sequence analysis algorithms.

## Introduction

Since the beginning of the 21st century, with the development of high-throughput sequencing technology, gene sequencing has become much cheaper and more efficient, enabling the development of various genome projects. Since the implementation of the Human Genome Project (Collins et al., 1998), the amount of bioinformatics data being produced has grown explosively, with genome sequencing data doubling every 4–5 months. At the same time, bioinformatics (Zhang, 2000), a new interdisciplinary subject, has developed rapidly. Bioinformatics covers all aspects of the acquisition, processing, storage, distribution, analysis, and interpretation of biological information. It integrates tools from mathematics, computer science, and biology to clarify and understand the biological significance of large amounts of data (Hogeweg and Searls, 2011). One of the major problems faced by bioinformatics today is how to process the data generated by genetic engineering. Data are not information; data need to be mined using systematic scientific methods to find biologically relevant information.

Sequence alignment is a fundamental method to study biological sequence data in bioinformatics (Mount, 2005). The theoretical basis of sequence alignment is the chemistry in biology, that is, if the similarity between two biological sequences reaches a threshold, it is considered that they have similar functions and structures as well as evolutionary relationships. By comparing an unknown biological sequence with a known functional structure, and identifying similar regions between them, the homology between the species can be judged, and the biological information contained in the unknown sequence can be revealed. According to the number of sequences to be aligned, sequence alignment can be divided into pairwise and multiple sequence alignment. The standard solution for pairwise sequence alignment is to use a dynamic programming algorithm to find the optimal solution. The classical algorithm is the Needleman-Wunsch (Needleman and Wunsch, 1970) algorithm, which is used to solve the global pairwise sequence alignment problem; the more biologically significant local alignment problem can be solved by the Smith-Waterman (Smith and Waterman, 1981) algorithm. Also, the heuristic-based BLAST (Altschul et al., 1990) algorithm is widely used in similarity sequence searches of gene databases. Theoretically, the dynamic programming approach to pairwise sequence alignment can be used for multiple sequence alignment problems. A two-dimensional dynamic programming matrix is extended to the three-dimensional or multi-dimensional case, where the dimension of the matrix reflects the number of sequences to be compared. This method is only suitable for multiple sequence alignments with few dimensions, otherwise it will be a great challenge with respect to computer resources. It has been proved that the multiple sequence alignment problem based on the SP (sum of pairs) metric is NP (Wang and Jiang, 1994), and multiple sequence alignment uses a heuristic algorithm. Here, we mainly focus on the heuristic multiple sequence alignment algorithm (HMSAA) domain.

HMSAAs include progressive alignment (Feng and Doolittle, 1987) and iterative alignment (Wang and Li, 2004); this paper mainly considers the progressive alignment method. The progressive multiple sequence alignment algorithm was proposed by Feng and Doolittle in 1987. Thompson and Higgins implemented the progressive multiple sequence alignment algorithm and proposed the ClustalW (Thompson et al., 1994) algorithm. Subsequently, Notredame et al. (2000) proposed the T-Coffee (tree-based consistency objective function for alignment evaluation) algorithm; the latter two algorithms are the most commonly used progressive multiple sequence alignment algorithms. The HAlign (Zou et al., 2015) algorithm is a progressive alignment algorithm based on central star alignment. Clustal Omega (Sievers et al., 2011) is a completely rewritten and revised version of the widely used Clustal series of programs for multiple sequence alignment. The main improvement over ClustalW algorithm is the use of the mBed algorithm to generate guide trees of any size and the use of HHalign Package based on the idea of hidden Markov model in the last step of Profile alignment. The main disadvantage of the progressive multiple sequence alignment algorithm is its principle of “once vacant, always vacant.” The errors generated in the alignment will always affect the sequence alignment process, which may lead to a suboptimal result and reduce the accuracy of the algorithm. The basic idea of the progressive alignment algorithm is that there is an evolutionary relationship between the multiple sequences that are aligned; after determining the evolutionary order of the sequences, they are gradually aligned along the evolutionary order until all sequences are aligned. This means that before proceeding to the progressive alignment, it is necessary to find the evolutionary relationship between the sequences. At present, optimization of the progressive alignment algorithm usually focuses on the step of confirming the evolutionary relationship (Zhang et al., 2005; Huo and Xiao, 2007). In order to speed up sequence alignment when the scale of the alignment is large, parallel computing may be combined with progressive alignment (Hung et al., 2015). The basic idea of iterative alignment is first to improve the multiple sequence alignment based on an algorithm that can generate alignments, through a series of iterations, until the alignment results no longer improve or have reached the maximum number of iterations. This paper mainly considers the combination of iterative alignment and progressive alignment. Such algorithms, which include MultAlin (Corpet, 1988) and Muscle (Edgar, 2004), have improved robustness and wider application scope.

At present, most research on sequence alignment algorithms focuses on the optimization of specific steps of a particular algorithm. The optimization effect on different sequences will be different, and the diversity and complexity of sequence alignment algorithms may make it difficult for users to select an algorithm appropriate to the characteristics of a given sequence, resulting in unnecessary computing errors in practice. On the other hand, it may be difficult for users to understand the structure of a sequence alignment algorithm, which may affect its correct use and to some extent affect the accuracy of the sequence analysis. The specificity and low abstraction of a sequence alignment algorithm reduce its reusability and maintainability. Therefore, it is necessary to study sequence alignment algorithms at the domain level. Concerns on algorithm families will be helpful for extracting the commonality and variability of different algorithms and for the formal development of sequence alignment algorithms.

In this work, the generative programming method is used to design an abstract generic algorithm component library, after which a specific alignment algorithm for the HMSAA domain is assembled, thus improving the reliability and reusability of the algorithms. First, domain analysis of HMSAA is carried out, the common domain features and variability features are identified, and a domain feature model of HMSAA is established. Furthermore, relationships among features are analyzed and an interaction model of algorithm components is designed and constructed. Finally, using a generic abstract programming language, Apla, the domain components are formally implemented and a high abstract component library is built on top of Apla.

## Related Methodology and Technology

### Generative Programming

Software reuse is considered to be one of the solutions to the “software crisis.” High-quality software reuse can improve the efficiency and quality of software development and ultimately result in the construction of an industrialization pipeline to develop software. Generative programming (Czarnecki and Eisenecker, 2000) is the use of components and the creation of software products in an automated manner. Implementation consists of two steps. First, the current software development model is transformed into the development of the software system family. Then, a generator is used to automatically assemble the components. Through domain analysis of the software system family, generative programming constructs a domain model of the system family and further develops the domain design and domain based on this model. New software development in the same field is based on the established domain model, and the reusable components are selected for assembly and implementation. It is not the development of software.

**Figure 1:**
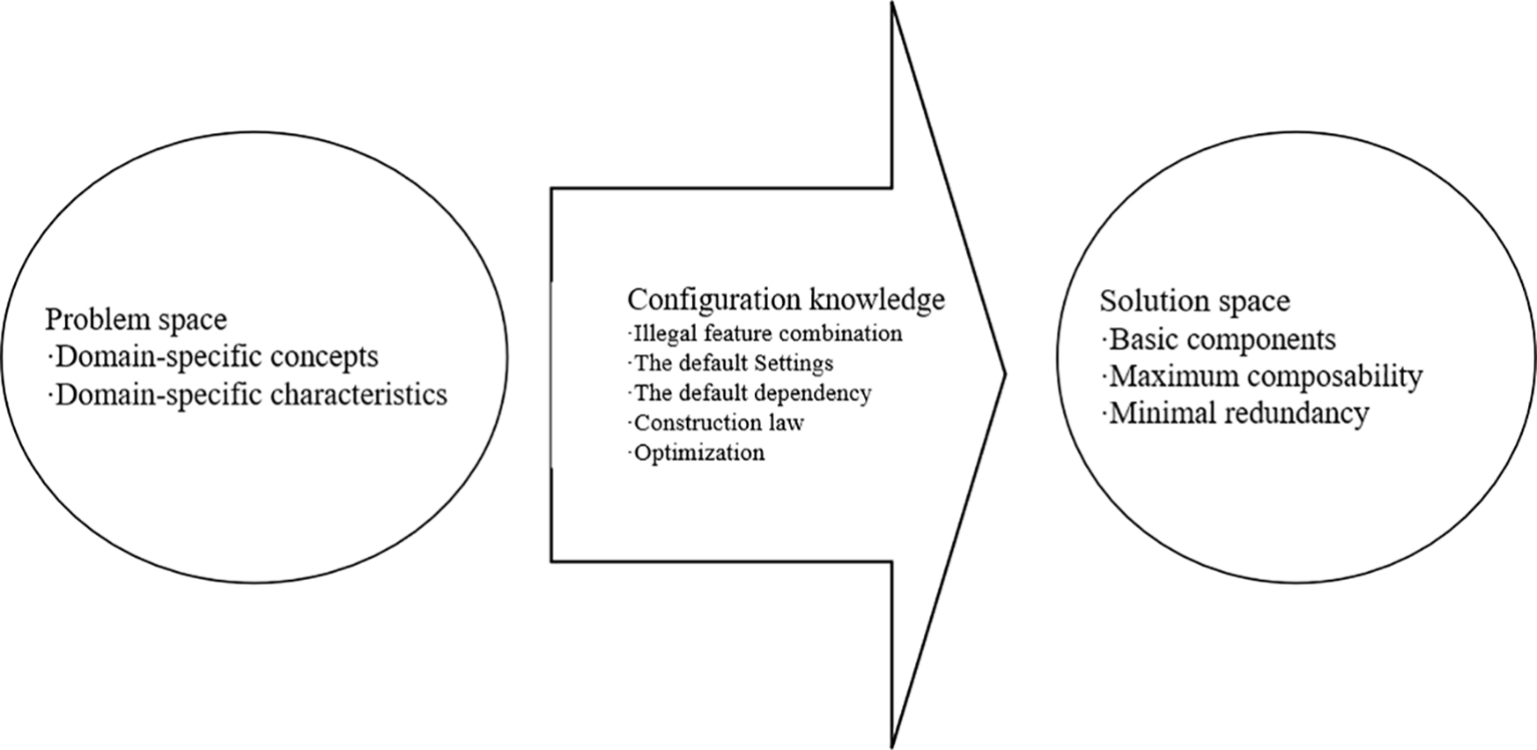
Composition of generative domain model.

A domain model based on generative programming includes a problem space, a solution space, and domain-specific configuration knowledge for mapping between the two. The problem space is used to represent the requirements of the customization system, and is mainly for use by application programmers and customers. The solution space includes the implementation components required for the system family implementation and the combination, dependencies, and interactions among implementation components. Domain-specific configuration knowledge is mainly used to separate the problem space and solution space, which not only reduces the redundancy and coupling of the implementation components but also improves their composability and reusability. The composition of such a generative domain model is shown in **Figure 1**.

### Domain Modeling

**Figure 2:**
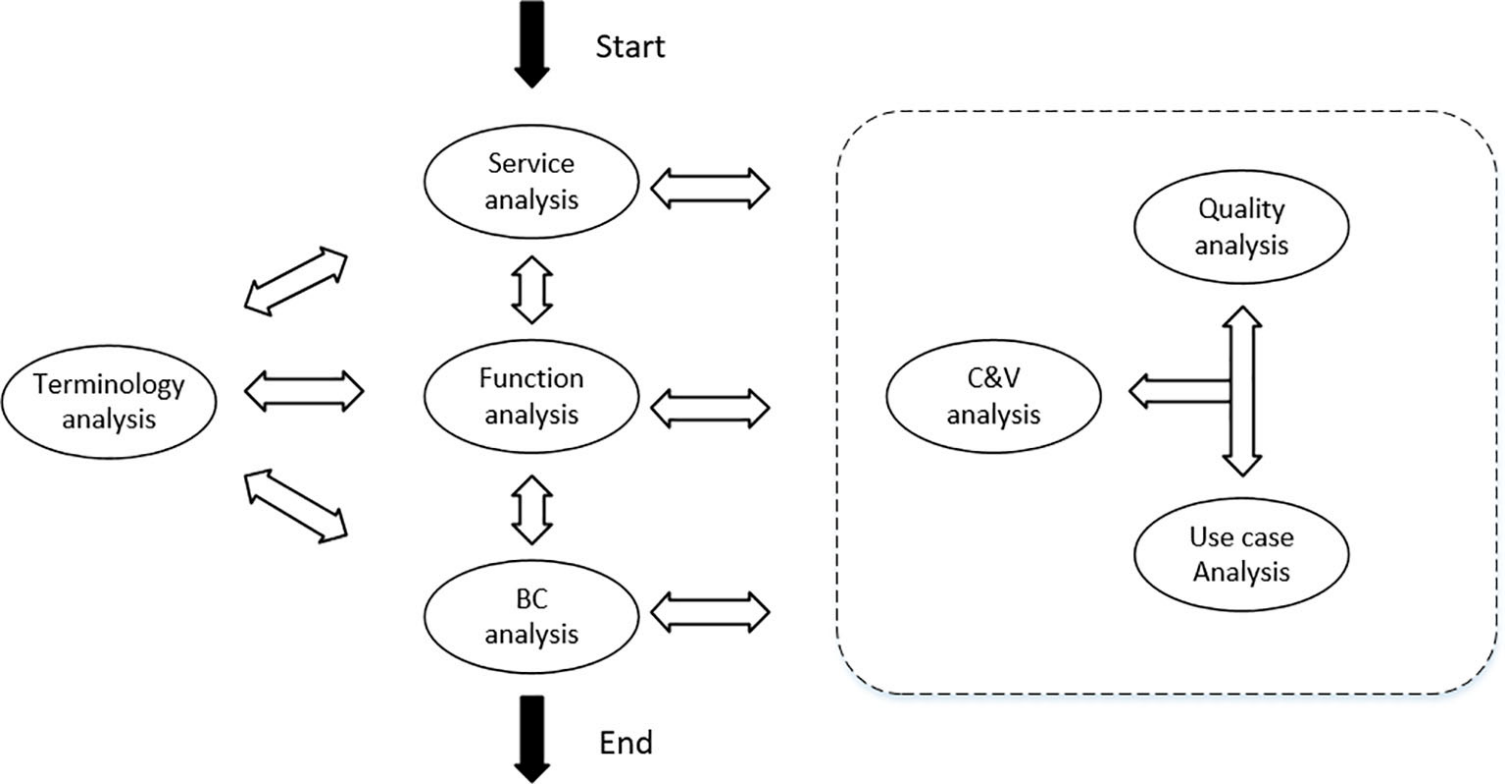
Feature modeling process.

Domain modeling requires the identification and modeling of key concepts (Lee et al., 2002). Feature engineering (Turner et al., 1998) considers features to be first-order entities that traverse the software life cycle and span the problem space and solution space, and reduce the difference in demand awareness between users and software developers through features. Features in FODA (feature-oriented domain analysis) (Kang et al., 1990) are considered to be user-visible, significant, and distinctive aspects, qualities, characteristics, *etc*., in a software system. Features are the domain knowledge accumulated by users and experts from long-term practice in a domain. Feature modeling is an activity that models the commonality and variability of features and the relationships among them. Zhang and Mei (2003) proposed a feature-oriented domain modeling (FODM) method that considered the features of services, functions, behavioral characteristics, *etc*. This was based on service analysis activities, functional analysis activities, and behavioral characteristics analysis in combination with domain terminology analysis, commonality and variability analysis, interactive process analysis, and quality demand analysis concurrently, with continuous retrospective refinement to finally obtain the feature model. The domain modeling process is illustrated in **Figure 2**.

### Partition and Recur Method

PAR (Xue, 1993; Xue, 1997; Xue, 1998; Shi and Xue, 2009; Xue, 2016) (partition and recur) is a formal development method based on partition and recursion, containing an algorithm design language (Radl; recurrence-based algorithm design language), an abstract generic programming language (Apla), and a unified algorithm design and proof method, as well as a series of generation systems (the PAR platform).

The Apla language can be used to directly write programs using abstract data types and abstract procedures. It has the advantages of concise and rigorous mathematical language, and its high level of abstraction makes it suitable for describing abstract algorithmic programs.

The generic programming mechanisms supported by Apla include type parameterization, subroutine parameterization, and user-defined generic abstract data types (ADT). 1) Apla introduces the keyword *sometype* to define the type variable, the type parameter, the parameter return value type of the procedure function, and the basic type of the combined data type. The type is used as a parameter to implement the generalization of the program. 2) Apla subroutine parameterization includes procedure parameterization and function parameterization. In a subroutine, the keywords *proc* and *func* are used to declare procedure parameters and function parameters, and the procedure or function is used as a parameter list. 3) As well as the predefined ADT in Apla, users can create custom ADT to make the language more flexible and the program description more powerful. These custom operations include the definition and the implementation of ADT. The ADT definition contains the operation name, the operation type, the parameters of the operation, *etc*. The ADT implementation gives the specific implementation methods of these operations, and *define*, *ADT*, *enddef*, *implement*, *endimp*, and other keywords are used to describe the custom ADT. In addition, the PAR platform supports the transformation of Apla into an executable high-level programming language such as C++ or Java.

## Heuristic Multiple Sequence Alignment Algorithm Modeling

**Figure 3:**
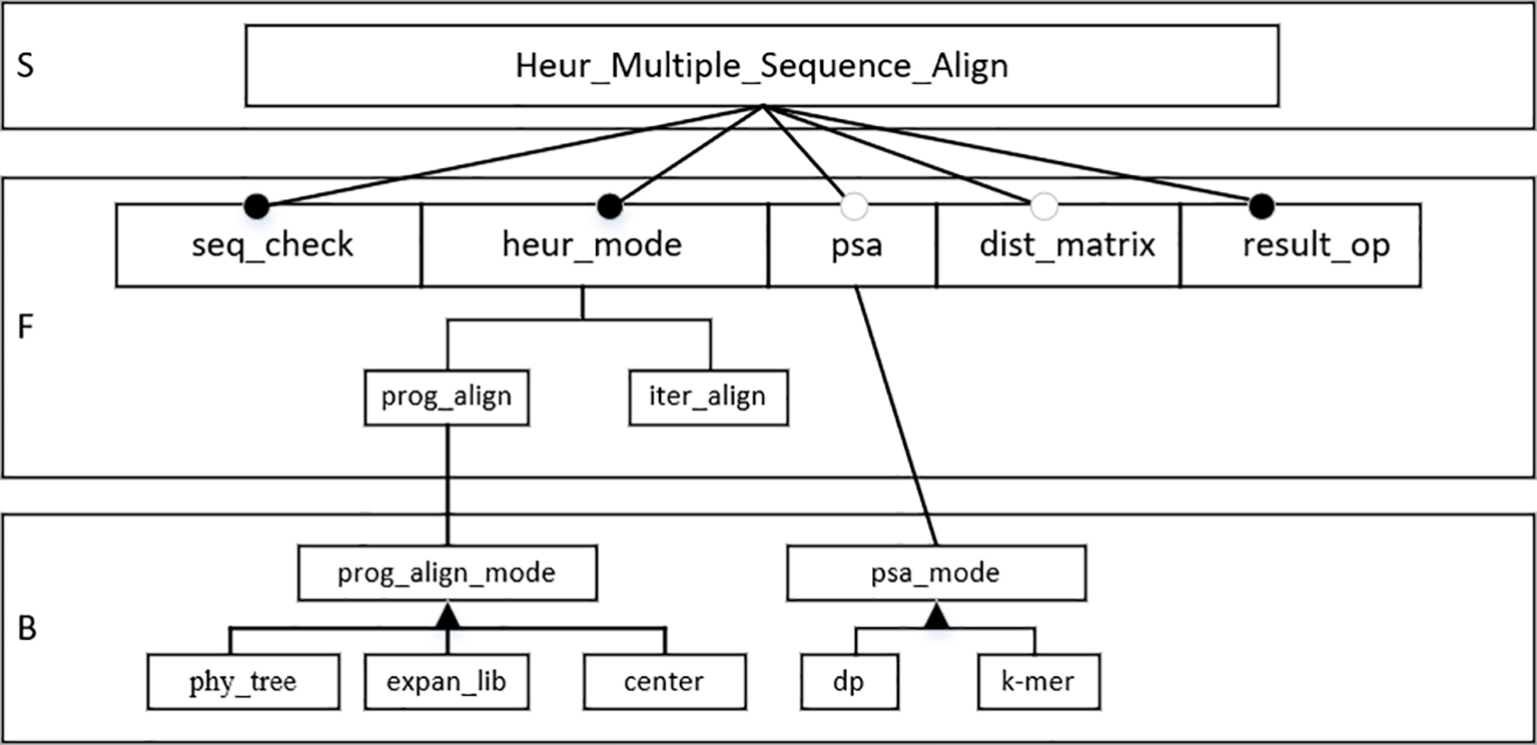
Feature model of heuristic multiple sequence alignment algorithm (HMSAA).

In this section, the FODM method is used to construct the feature model according to the service, function, and behavior characteristics in the HMSAA domain. Heuristic multiple sequence alignment operations are core services in the domain. The sequence legality check (*seq_check*), heuristic alignment mode selection (*heur_mode*), pairwise sequence alignment operation (*psa*), distance matrix (*dist_matrix*), result output (*result_op*), progressive alignment (*prog_align*), and iterative alignment (*iter_align*) are the main functions in the domain. Progressive alignment and iterative alignment are sub-functions of heuristic alignment mode selection. Sequence legality check, heuristic alignment mode selection, and alignment result output are mandatory, where as function, pairwise sequence alignment operation, and distance matrix are optional. For progressive alignments, the progressive alignment mode *(prog_align_mode*) is a behavioral feature that has the following three values: the phylogenetic tree (*phy_tree*), the extended library (*expan_lib*), and the center alignment. For pairwise sequence alignment operations, the pairwise sequence alignment mode (*psa_mode*) is a behavioral feature that has two values, fast alignment (*k-mer*) and dynamic programming alignment (*dp*). According to the above analysis, a feature model was constructed for the domain, as shown in **Figure 3**.

## Design and Implementation of Heuristic Multiple Sequence Alignment Algorithm Components

### Interaction of Algorithm Components in Heuristic Multiple Sequence Alignment Algorithm Domain

According to the feature model described in the previous section, in order to achieve a complete library of algorithm components, it is necessary to further analyze the interaction modes among different algorithm components. The interactions of algorithm components involve constraints and dependencies between features. Therefore, this section describes an interaction model of algorithm components in the HMSAA domain according to their interaction modes.

**Figure 4:**
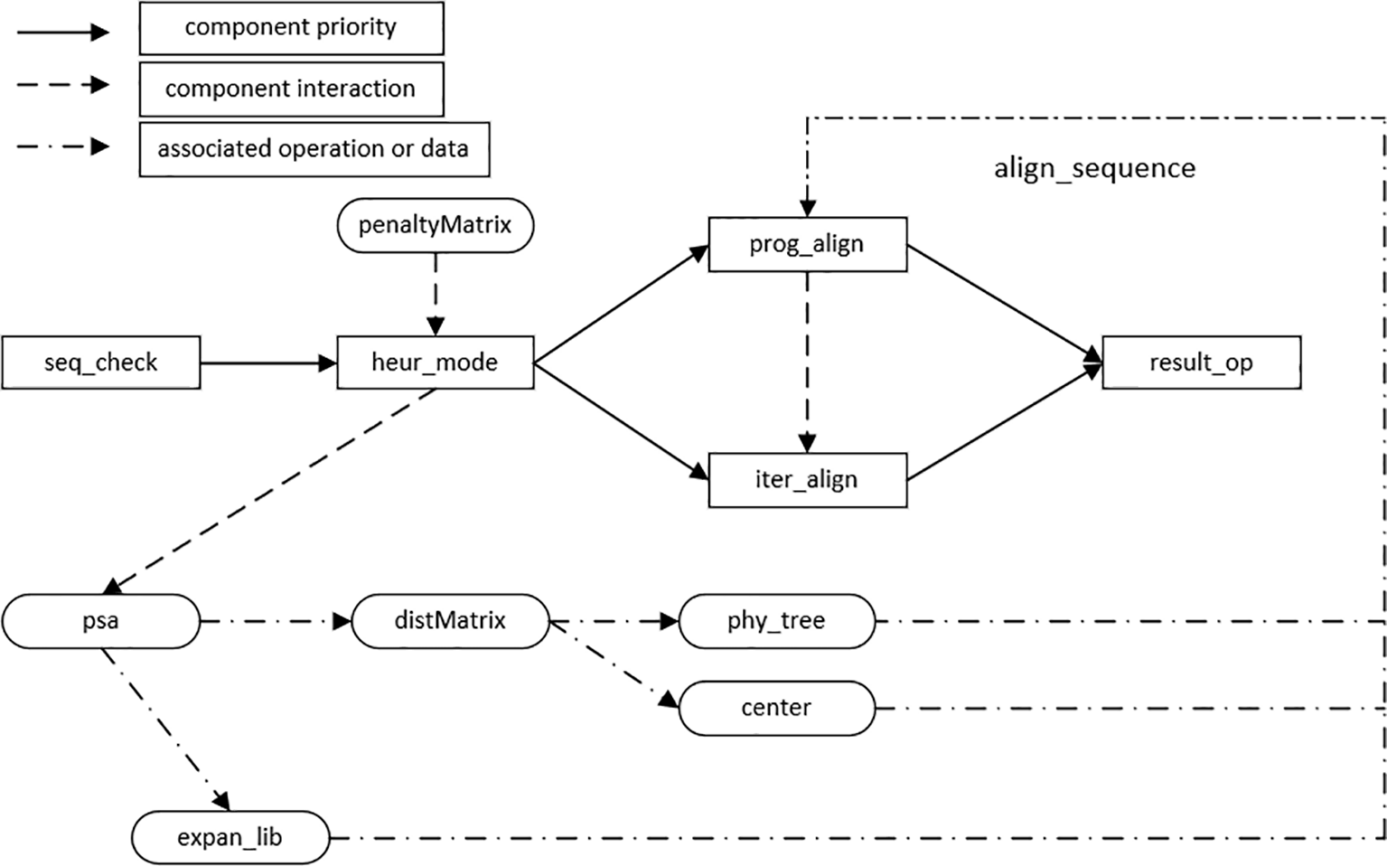
Algorithm components interaction model.

Through the establishment of the HMSAA feature model, it can be concluded that the algorithm consists of four main process features, i.e., heuristic alignment mode selection, progressive alignment, iterative alignment, and result output. In addition, the input of the algorithms in this domain consists of sequences of biological information, including DNA, RNA, and protein sequences. Before the implementation of the algorithm, the legality of the sequence information needs to be checked, for example, a DNA sequence can only contain four letters, *A*, *T*, *C*, and *G*. The main components in this domain are sequence legality checking, heuristic alignment mode selection, progressive alignment, iterative alignment, and result output. Other features and data structures in the feature model are used as auxiliary components, and the interaction model of components is established according to the dependencies between them, as shown in **Figure 4**.

The nodes connected by solid lines are the basic components that must be included in the HMSAA domain, namely, the three mandatory features and two sub-features selected by the heuristic mode. The solid line arrow denotes the execution priority of the component from high to low along the direction of the arrow.

Dotted and underlined arrows represent the data, structures, and associated operations required in the assembly of the algorithm components. For example, the establishment of an extended library requires the information of alignment in *psa*. The dotted arrow indicates the interaction between the two components during the execution of the algorithm. For example, before an iterative alignment, a progressive alignment should be carried out. By setting the number of iterations and the iterative method, the result of the progressive alignment is iterated until there is no further change or the maximum number of iterations has been reached.

In summary, the above interaction model includes most mainstream HMSAAs, including the progressive alignment algorithm based on tree and central alignment, the progressive alignment algorithm based on a compatible optimization objective function to form an expanded library, and the multiple sequence alignment algorithm combining iteration and progress. Here we just outline simple formal specification description of two components below for examples.

1 seq_check component

|[**in** a[][]: Array[][]; **out** bl: Boolean]|
AQ: bio-sequences.
AR: if *bl* is true, the legality check is passed; false is the opposite.

2 prog_align component

|[**in** heur_mode: ADT; phy_tree: ADT; a[][]: Array[][] **out** b[][]: Array[][]]|
AQ: phy_tree component, the sequence to be aligned, heur_mode component.
AR: alignment of multiple sequences.

Here *in* and *out* in the front of pre-condition *AQ* are two key words defined in PAR platform and are used to denote the input and output respectively; array, *Boolean*, etc., are the predefined types in PAR platform, and AR stands for post-condition of algorithm.

### Apla Formal Implementation

In this section, we make use of the advantages of Apla, including high-level abstraction, strong support for ADT, and easy correctness verification, and formally implement the HMSAA model. Here, only the implementation of the tree-based progressive alignment algorithm is illustrated.

1 seq_check component

Check whether the sequence group meets the biological definition. For example, the character set of the DNA sequence is {A, T, C, G}.

  procedure *seq_check*(*a[]*:array[String]);

2 Penalty component

We designed the penalty model as an ADT, using an affine penalty model, where *sometype* is a keyword in the Apla language that defines the type variable. *GapOpen*, *GapExtend*, and *score* represent the penalty points of open vacancy, extended vacancy, and non-vacancy, respectively.

  define ADT *penaltyMatrix*(sometype*elem*);
  GapOpen : Integer;
  GapExtend : Integer;
*  score*:array[array[Integer]];
  enddef.

3 heur_mode component

The *Heur_mode* component is defined as an ADT that selects the operation mode of multiple sequence alignment and defines the data structure and information required for alignment. The *setPenaltyMatrix*, *setGapOpen*, and *setGapExtend* functions are to set penalty matrix, open vacancy penalty, and extend vacancy penalty, respectively. The generic procedure *tree_prog_align* sets the alignment mode to one designated by the user. The useLib means to select the expan-lib component, useFullPW denotes the use of the conventional dynamic programming pairwise sequence alignment algorithm, useIter represents the iteration, and treeAlgorithm is the algorithm to generate the phylogenetic tree.

define ADT*heur_mode*(sometype*elem*);
function *setPenaltyMatrix*(*pm*: penaltyMatrix):Array[Array[Integer]];
function*setGapOpen*(*gapOpen*: Integer): Integer;
function *setGapExtend*(*gapExtend*:   Integer): Integer;
procedureprog_*align*(useLib: bool; useFullPW: bool; useIter: bool; treeAlgorithm : String)
enddef.

4 dist_matrix component

The *dist_matrix* is defined as an ADT that calculates the distance matrix element and returns it using the score of the pairwise alignment, and the pairwise sequence alignment operation is defined as the generic parameter. The function *getDist* is used to get the data from the distance matrix. Proc psa is described in detail in reference (Shi and Zhou, 2019).

define ADT dist_matrix (sometypeelem);
  function *calDistMat* (proc *psa*(…):Array[Array[Integer]];//.
  function getDist (ii: Integer; jj: Integer): Integer;
······
enddef;

5 phy_tree component

*Phylotree* data structure is defined as an ADT, facilitating subsequent operations on the tree. The parameters *treeMess*, *left*, and *right*, respectively, represent the information of the tree node and the left and right subtrees. The *phylotree* component is defined as an ADT that generates a phylogenetic tree using the data in the distance matrix. The ADT contains the generic procedure *generateTree* and takes *selAlgorithm* as its generic parameter. The generic procedure can generate phylogenetic trees through different algorithms. The function *calWeight* calculates the weight of each sequence when calculating the score of multiple sequences alignment, the generic procedure *getStepsForMSA* is used to generate the sequence of subsequent multiple sequence alignments, and the generic procedure *readTree* is used to read information from the generated phylogenetic tree.

define ADT *phyloTree* (sometype*elem*);
  *treeMess*: Array[Array[Integer]];
*  left*: Integer[];
*  right*: Integer[];
enddef.
define ADT *phy_tree* (sometype*elemMatrix*);
  procedure *generateTree*(*distMat*: elemMatrix; *seqName*: String[]; func*selAlgorithm():String*;treeName : String; *result*: Boolean);
  function *calWeight* (*firstSeq*: Integer; *lastSeq*: Integer; *seqsW*: Array[Integer]): Array[Integer];
  procedure *readTree* (*seqName*: String[]; *treeName*: String; *firstSeq*: Integer; *lastSeq*: Integer);
  procedure *getStepsForMSA* (proc*readTree*; *distMat*: elemMatrix; *result*: Boolean);
······
enddef.

6 prog_align component

The *prog_align* component is defined as an ADT that includes the generic procedure *multiSeqAlign*, which performs progressive alignment according to the alignment order obtained from the phylogenetic tree and the sequence weight.

define ADT *prog_align* (sometype*elem*);
 procedure multiSeqAlign (*seqs*: Array[String]; *steps*: elem; *seqName*: String[]; *seqW*:Array[Integer]; *start*: Integer);
 ······
enddef.

7 result_op component

The *result_op* component is defined as an ADT. It is composed of two generic procedures, *multiAlign_op* and *phyloTree_op*. The *multiAlign_op* procedure annotates the results of multiple sequence alignments and outputs them; *pathAlignOutput* is the path of the output file. The p*hyloTree_op* procedure outputs the phylogenetic tree; here, *pathTreeOutput* is the path of the output file.

define ADT *result_op*(sometype*elem*)
 procedure *multiAlign_op* (*pathAlignOutput*: String; *seqs*: Array[String]; *seqName*: String[]; sometype*prog_align*);
 procedure *phyloTree_op* (*pathTreeOutput*: String; *seqName*: String[]; sometype*phyTree*; sometype*distMat*);
enddef.

### Assembly of Clustal Algorithm

In this section, a phylogenetic tree-based progressive alignment algorithm, *clustalW*, is assembled on top of the HMSAA component library introduced in previous section. The Apla program is as follows.

program clustalW;
const/* input sequences*/
var
seqs, seqsName: Array[String];//Seqs is the sequence to be aligned
  //seqsName is the identification name of the sequence
const pathTreeOutput, pathAlignOutput: String;
/*omit the initialization of pairwise sequence alignment*/
ADT pm: new penaltyMatrix ();
ADT psa: new psa (……);
ADT distM: new dist_matrix (psa);
ADT phyloTree: new phylotree ();
ADT tree: new phy_tree (phyloTree; distM);
ADT msa: new prog_align (tree);
ADT mode: new heur_mode (pm);
var
clustalw: mode; gapOpen, gapExtend: Integer; penalty: Array[Array[Integer]];
begin
clustalw.setPenaltyMatrix (penalty);
clustalw.setGapOpen (gapOpen);
clustalw.setGapExtend (gapExtend);
end;
ADT resultOp: new result_op ();//instantiate and initialize the required components
procedure heur_multiple_sequence_align (clustalw; psa; distM; tree; msa; resultOp);
//heuristic multiple sequence alignment operations
var
NJTree: String; result: Boolean
begin
 check (seqs);
clustalw.prog_align (false; true; false; “NJTree”);
if(clustalw.getUseLib = false)→
if(clustalw.getUseFullPW = true) →
distM.calDistMat (psa);
tree.generateTree (clustalw.getTreeAlgorithm; seqsName; clustalw; distM;
result);
msa.multiSeqAlign (seqs; seqsName; tree; 0);
resultOp (msa; tree; pathTreeOutput; pathAlignOutput);
end.

## Experiments

As the Apla language cannot run directly, in this section we make use of the PAR platform to transform the Apla algorithm components into the corresponding C++ components.

**Figure 5:**
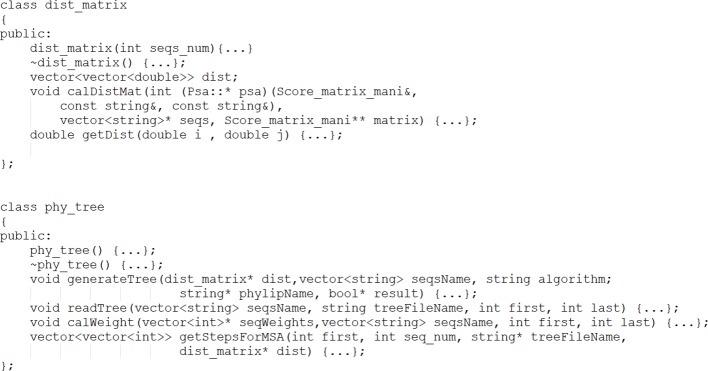
Result of ADT transformation.

ADT algorithm components in Apla containing only data members are transformed into *struct* data types in C++, such as *penaltyMatrix* and *phyloTree*. The results are as follows.

struct penaltyMatrix
{
  int gapOpen;
  int gapExtend;
  vector < vector < int>> score;
};
struct phyloTree
{
  vector < vector < int> > treeMess;
  vector < double > leftBranch;
  vector < double > rightBranch;
};

ADT components containing data members and member functions are transformed into classes in C++, such as *dist_matrix* and *phy_tree*. The function body code is long, and so part of it is omitted here. The partial result of the transformation is shown in **Figure 5**.

**Figure 6:**
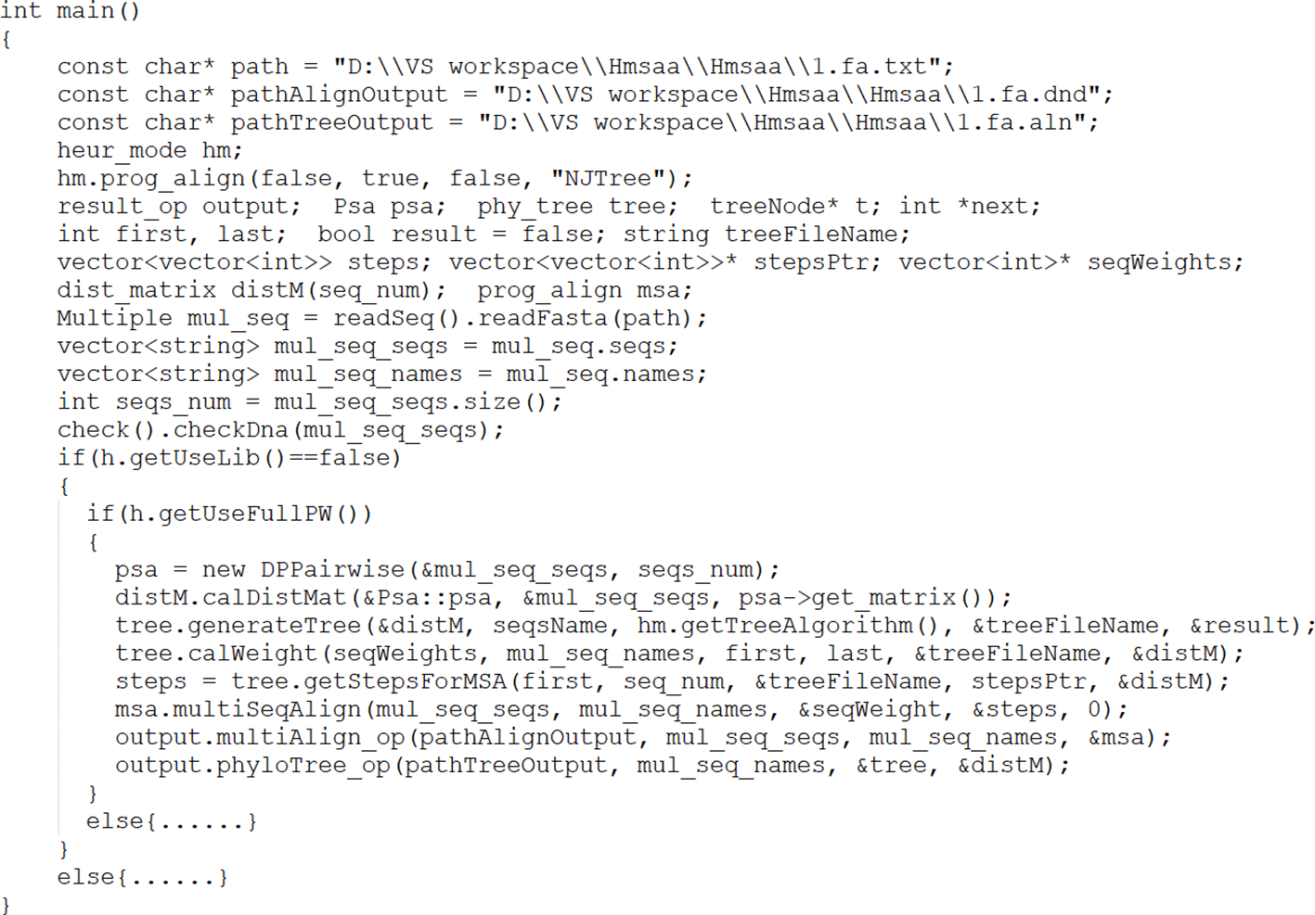
C++ assembly process of clustalW.

Generic procedures and functions defined in Apla are converted into separate class member functions in C++ to reduce coupling between components. In particular, the calling functions are converted into indicator functions in C++, and the generic parameter is converted into the pointer parameter to implement the polymorphism of the Apla program. After converting each component into C++, the Apla code for the heuristic multiple sequence alignment operation is converted into the main function executed in C++; finally, the *clustalW* algorithm program is run through manual assembly of the components, as shown in **Figure 6**.

**Figure 7:**
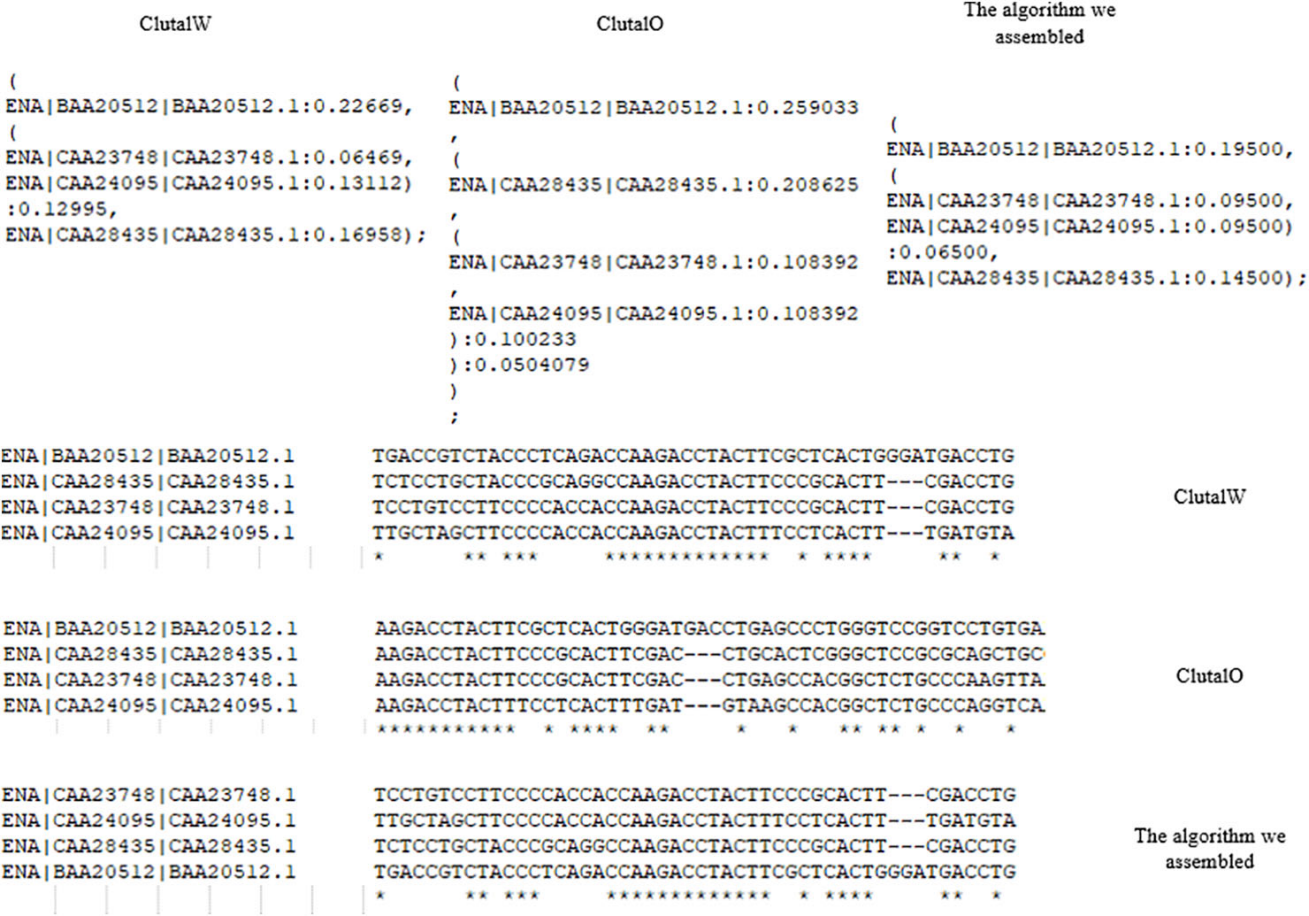
Alignment results.

To test the program, we used four pieces of DNA data, *Cyprinus carpio* (common carp) alpha-globin, *Homo sapiens* (human) alpha globin, *Mus musculus* (house mouse) alpha-globin, *Capra hircus* (goat) alpha-globin. The alignment results between our algorithm and the other two Clustal algorithms are shown in **Figure 7**. Due to the different selected pairwise alignment parameters and types, the structure of the phylogenetic tree is different from that of the ClutsalW algorithm, and the sequence of alignment has also changed, but the results remain biologically significant.

## Summary and Future Work

As a key topic in bioinformatics research, sequence alignment algorithm and its applications have attracted extensive research attention. However, there has been no work considering it as a domain for high-level abstraction to improve the reliability and the productivity of the algorithms, and to reduce the probability of suboptimal solutions, errors of the algorithm, etc.

Generative programming and the composition of a generative domain model are first briefly presented in the paper, and the FODM method is described. The feature model can be obtained by taking the characteristics of the service, function, and behavior characteristics of the domain into account, and carrying out a series of feature analysis activities.

By using generative programming and feature modeling, the HMSAA domain has been analyzed, resulting in the following three algorithm classes: progressive alignment algorithms based on tree and central alignment; progressive alignment algorithms based on the compatible optimization objective function to form an expansion library; and multiple sequence alignment algorithms based on a combination of iteration and increment. Through analysis of this domain, general and variable features have been extracted and mapped to components, and an HMSAA feature model has been established. Moreover, an interaction model of HMSAA domain components has been designed based on the relationships among features and formally implemented using the generic abstract programming language Apla in support of the PAR platform. An HMSAA component library has been established, the reliability of which can be guaranteed owing to the ease of verification with the Apla language.

It is expected that the formal components could be automatically or semi-automatically assembled to generate a specific problem-solving algorithm, thus reducing the errors resulting from manual algorithm selection for multiple sequence alignment, and improving the algorithm efficiency, which will enable assembly of a new, more efficient, multiple sequence alignment algorithm. Furthermore, the high-level abstraction of generic components, such as *generateTree*, provides a diversity of algorithm components assembly as well as a good demonstration of the connections between algorithm features, thus improving the understandability and ease-of-use of algorithms.

Next, we will release our codes in GitHub. Future work also include developing a user-friendly visual interface to facilitate component assembly. Users will be able to generate different sequence alignment algorithms by selecting different components *via* the interface and use XML files to describe the composition and constraint relations among components, without any change to the component library. We are encouraged by the success of algorithm assembly on the PAR platform.

The methodology and techniques for HMSAA are not only applicable to multiple sequence alignment algorithms but also have theoretical reference significance and practical application value for other biological sequence analysis algorithms, such as the assembly algorithm based on DeBruijn graph structure used in the process of gene assembly (Li et al., 2010; Peng et al., 2012).We are currently applying some of these ideas to more problems in the domain of biological sequence analysis, to implement automatic or semi-automatic assembly of an algorithm component library based on the PAR platform. We hope to report on this work in the near future.

## Data Availability Statement

The datasets generated for this study are available on request to the corresponding author.

## Author Contributions

HS instructed the whole research work and revised the paper. XZ did the codes work and the experiments. All authors read and approved the final manuscript and are agree to be accountable for all aspects of the work.

## Funding

This work was supported by the National Natural Science Foundation of China under Grant Nos.61662035, 61762049, and 61862033.

## Conflict of Interest

The authors declare that the research was conducted in the absence of any commercial or financial relationships that could be construed as a potential conflict of interest.
